# 

**Published:** 1896-03

**Authors:** 


### CONTENTS.

ORIGINAL ARTICLES—

Urinary Analysis. Louis H. Jones, A. M., M. D., Atlanta, Ga. 105

The Endemic Fever of the Eastern Shore. By Geo. W. LeCato, M. D., Wachapreague, Accomac County, Va. ................ 111

Chloroform. By Thos. R. Evans, M. D., Mt. Carbon, W. Va.... 121

Double Optic Neuritis. By J. M. Krim, M. D., Louisville, Ky.. 123

An Improved Syringe for Infiltration Anæsthesia ............ 127

SOCIETY REPORTS—

Clinical Society of Maryland .......... 129

BOOK REVIEWS .......... 138

EDITORIAL—

The History of Anæsthesia .......... 140

NOTES .......... 133

SPECIAL NOTES .......... 146

Prescriptions .......... 154

